# Combined analysis of VEGF and EGFR predicts complete tumour response in rectal cancer treated with preoperative radiotherapy

**DOI:** 10.1038/sj.bjc.6604172

**Published:** 2008-01-08

**Authors:** I Zlobec, T Vuong, C C Compton, A Lugli, R P Michel, S Hayashi, J R Jass

**Affiliations:** 1 Institute of Pathology, University Hospital of Basel, Schönbeinstrasse 40, Basel 4031, Switzerland; 2 Department of Radiation Oncology, McGill University Health Centre, 1650 Cedar Avenue, Montreal, QC, Canada H3G 1A4; 3 Office of Biorepositories and Biospecimen Research, National Cancer Institute, National Institutes of Health, 31 Center Drive, Bethesda, MD 20892, USA; 4 Department of Pathology, McGill University, 3775 University Street, Montreal, QC, Canada H3A 2B4; 5 Department of Pathology, Toyama University, 2630 Sugitani, Toyama 930-0194, Japan; 6 Department of Cellular Pathology, St Mark's Hospital, Imperial College London, Watford Road, Middlesex HA1 3UJ, UK

**Keywords:** rectal cancer, VEGF, EGFR, immunohistochemistry, pre-operative radiotherapy, brachytherapy

## Abstract

The ability to predict complete pathologic response or sensitivity to radiation before treatment would have a significant impact on the selection of patients for preoperative radiotherapy or chemo-radiation therapy schedules. The aim of this study was to determine the value of epidermal growth factor receptor (EGFR), vascular endothelial growth factor (VEGF), p53, Bcl-2 and apoptosis protease-activating factor-1 (APAF-1) as predictors of complete pathologic tumour regression in patients undergoing preoperative radiotherapy for advanced rectal cancer. Pretreatment tumour biopsies from predominantly cT3 patients undergoing a preoperative high-dose-rate brachytherapy protocol were immunostained for EGFR, VEGF, p53, Bcl-2 and APAF-1. Immunoreactivity was evaluated by three pathologists. Cut-off scores for tumour marker positivity were obtained by receiver-operating characteristic (ROC) curve analysis. The association of marker expression with complete pathologic response was analysed in univariate and multivariable analysis. Multi-marker phenotypes of the independent protein markers were evaluated. In multivariable analysis, loss of VEGF (*P*-value=0.009; odds ratio (OR) (95% CI)=0.24 (0.08–0.69)) and positive EGFR (*P*-value=0.01; OR (95% CI)=3.82 (1.37–10.6)) both demonstrated independent predictive value for complete pathologic response. The odds of complete response were 12.8 for the multi-marker combination of VEGF-negative and EGFR-positive tumours. Of the 34 EGFR-negative- and VEGF-positive cases, 32 (94.1%) had no complete pathologic response. The combined analysis of VEGF and EGFR is predictive of complete pathologic response in patients undergoing preoperative radiotherapy. In addition, the findings of this study have identified a subgroup of simultaneous EGFR-negative and VEGF-positive patients who are highly resistant to radiotherapy and should perhaps be considered candidates for innovative neoadjuvant combined modalities.

Colorectal cancer is a leading cause of cancer-related mortality and morbidity in the Western world, with 5-year survival rates ranging from 90 to 10% with tumour progression (O'Connell *et al*, 2004). Patients with rectal cancers, comprising approximately 30% of these cases, are known to have an increased rate of local recurrence and decreased survival time compared with patients with tumours of the colon, a result due primarily to the surgical constraints imposed by the location of the rectum within the pelvis (Wolpin *et al*, 2007). As a consequence, the clinical management of patients with rectal cancer differs significantly from that of the colon in terms of surgical technique, the more frequent use of radiotherapy and method of chemotherapy administration.

Evidence from randomised clinical trials, meta-analyses and epidemiologic studies strongly support the treatment of rectal cancer with preoperative modalities (Colorectal Cancer Collaborative Group, 2001; Wong *et al*, 2007). The Swedish Rectal Cancer Trial was the first to demonstrate an independent survival benefit and significant improvement in local control with preoperative short-course radiotherapy (25 Gy delivered in five fractions over 1 week) compared with surgery alone (Swedish Rectal Cancer Trial Group, 1997). Similar findings were derived from the Stockholm II Trial with the same fractionation regimen (Martling *et al*, 2001). Recently, short-course preoperative radiotherapy was found to decrease these rates even further in combination with total mesorectal excision (TME) (Kapiteijn *et al*, 2001). Short-course continuous, hyperfractionated, accelerated radiation therapy (CHART) and conformal high-dose-rate endorectal brachytherapy (HDREB) have demonstrated high rates of complete pathologic response as well as acceptable toxicity levels and tolerable side effects (Vuong *et al*, 2002, 2007; Brooks *et al*, 2006).

Several pathological features have been identified as prognostic factors in patients undergoing preoperative radiotherapy with or without chemotherapy. The circumferential resection margin status significantly affects prognosis with 5-year overall- and recurrence-free survival, decreasing substantially with increasing R-stage (den Dulk *et al*, 2007; Eriksen *et al*, 2007; Larsen *et al*, 2007). Rödel and co-workers investigated the tumour regression grade (TRG) on 5-year disease-free survival in more than 350 patients (Rodel *et al*, 2005). An independent adverse prognostic effect was observed in patients with low TRG. Kuo *et al* (2007) noted an improved survival in patients with complete pathologic response and a decreased prognosis in patients with more advanced post-treatment TNM stage. Das *et al* (2007) observed an association between complete pathologic response and loco-regional control.

In addition to these pathological prognostic factors, which can only be identified post-surgically, molecular characterisation is expected to improve the identification of more aggressive or treatment-resistant tumours before therapy. Recently, microarray gene expression profiling was successfully used to predict complete responses to preoperative chemoradiotherapy with advanced-stage rectal cancer (Ghadimi *et al*, 2005; Kim *et al*, 2007). Epidermal growth factor receptor (EGFR), vascular endothelial growth factor (VEGF), the p53 tumour suppressor and key mediators of cell-cycle arrest (p21, p27) and apoptosis (Bcl-2, apoptosis protease-activating factor-1 (APAF-1)) are among the immunohistochemical protein markers currently of interest as potential predictors of pathologic response, prognosis and recurrence-free survival in rectal cancer following neoadjuvant therapy (Giralt *et al*, 2005, 2007; Lopez-Crapez *et al*, 2005; Kim *et al*, 2006; Lin *et al*, 2006; Smith *et al*, 2006; Bertolini *et al*, 2007).

The aim of this study was to predict complete pathological tumour regression to preoperative HDREB by investigating the combined immunohistochemical expression of EGFR, VEGF, p53, Bcl-2 and APAF-1 in 104 pretreatment biopsies from patients with advanced rectal cancer.

## MATERIALS AND METHODS

### Preoperative HDREB

This study was approved by the Research Ethics Committee of the McGill University Health Center. One hundred and four patients with newly diagnosed invasive, resectable rectal adenocarcinoma were included in this study and informed written consent was obtained. Preoperative staging was performed according to the International Union against Cancer Classification and carried out by endorectal ultrasonography and magnetic resonance imaging (MRI). Eligible patients included those with large T2 tumours located in the middle 1/3 of the rectum, T3 and early T4 tumours. Patients with abdominal nodal disease, metastases and small T2 tumours with favourable features were excluded from the study. Tumour sizes ranged from 3 to 5 cm in diameter. Radiation was delivered preoperatively with a multi-channel endorectal applicator (Novi Sad and recently with the Oncosmart Nucleotron B.V., Veenendaal, Netherlands) and a high-dose-rate remote after-loading system using an Iridium-192 source (Vuong *et al*, 2007). A daily fraction of 6.5 Gy was administered over four consecutive days up to a total of 26 Gy. Each patient was planned with endorectal applicator in place using a CT simulator (Pickler International Inc., Highland Heights, OH, USA) in order to obtain optimal conformal dosimetry. The dose was prescribed to a clinical target volume that included the gross tumour volume and any intramesorectal deposits visible at MRI. Patients underwent surgery 4–8 weeks after brachytherapy as planned before treatment regardless of tumour response.

The assessment of tumour response was performed by pathologic evaluation of rectal specimens postoperatively. Tumours considered to be completely responsive to preoperative HDREB had no histologic evidence of residual viable carcinoma (ypT0). Tumours with microfoci, foci or large areas of residual carcinoma were considered partially or non-responsive to treatment.

### Immunohistochemistry

Immunohistochemistry for EGFR, VEGF, p53, Bcl-2 and APAF-1 was carried out on formalin-fixed, paraffin-embedded serial sections cut at 3 μm and dried at 37°C overnight. Immunostaining was performed using the avidin–biotin complex (ABC) procedure, including heat-induced epitope retrieval and enzymatic antigen retrieval procedures. Incubation was carried out overnight at 4°C for Bcl-2 (clone 124; DAKO, Glostrup, Denmark, 1 : 100) and VEGF (VEGF-A20; Santa Cruz Biotechnology, Santa Cruz, CA, USA, 1 : 100), and in a moist chamber at 37°C for 1 h for p53 (clone DO-7; DAKO, Denmark, 1 : 100) and APAF-1 (NCL-APAF-1; Novocastra, Newcastle, UK, 1 : 100). Immunohistochemistry for EGFR (clone 3C6, 3 mg ml^−1^; Ventana Medical Systems, Tucson, AZ, USA) was performed using an autostainer according to manufacturer's instructions. Negative controls were treated identically, with primary antibodies omitted.

### Evaluation of immunohistochemistry

Immunoreactivity was evaluated in a semi-quantitative manner from pretreatment biopsy specimens. The proportion of immunoreactive tumour cells over the total number of tumour cells by 5% increments (0, 5, 10, and so on up to 100%) was determined by three pathologists (A Lugli, JR Jass, S Hayashi) for EGFR and by four pathologists (CC Compton, A Lugli, JR Jass, RP Michel) for p53, Bcl-2, VEGF and APAF-1. This scoring method was previously found to be highly reproducible between pathologists (Zlobec *et al*, 2006a, 2007c). Only areas of invasive carcinoma were analysed. Protein expression was not evaluated in biopsies lacking sufficient tissue for immunohistochemical evaluation. Staining was assessed in the nucleus for p53 and in the cytoplasm for VEGF, Bcl-2 and APAF-1. Immunoreactivity for EGFR expression was assessed in both cytoplasm and/or membrane. Staining intensity was not evaluated.

### Statistical analysis

#### Selection of cut-off scores for protein positivity

Relevant cut-off scores for tumour positivity for each protein marker were obtained by performing receiver-operating characteristic (ROC) curve analysis (Zlobec *et al*, 2006b). Briefly, plots of sensitivity and (1-specificity) for complete pathologic tumour response were obtained for each marker and the (0,1)-criterion was used to select the threshold value, or protein expression score, above which expression was to be considered ‘positive’ (Bewick *et al*, 2004). In order to determine the reliability of the ROC curve-derived cut-off score, resampling of the data using 100 bootstrapped replications was performed for all proteins. To determine the discriminatory power of each marker for complete pathologic response, the area under the ROC curve (AUC), standard error (s.e.) and 95% CI were obtained for each. The closer the AUC to 1.0 is, the greater the predictive power of the marker for complete tumour response.

### Association with clinicopathological features at the respective cut-offs

The association of complete tumour response with both clinicopathological features and protein expression was analysed using logistic regression and where appropriate, with Fisher's Exact test. The *P*-values, odds ratio (OR) and 95% CI for each analysis were obtained. A Bonferroni correction for multiple comparisons was performed. To maintain an overall type I error rate of 0.05, each test of association with complete pathologic response was considered significant if *P*<0.005. All variables significant in univariate analysis were entered into a multiple logistic regression model. Statistical interactions between significant variables were tested. All analyses were carried out with SAS V9 (The SAS Institute, Cary, NC, USA).

## RESULTS

### Clinicopathological features

Patient characteristics are summarised in Table 1. Thirty-three (31.7%) patients had a complete pathologic tumour response to preoperative HDREB. Seventy-one (68.3%) were found to have residual carcinoma, including 35 patients (34.3%) who were considered as partial responders due to the presence of microfoci of residual carcinoma. Ninety-six (94.1%) patients were preoperatively staged as cT3. pT stage was available for 80 patients, of which 31 (38.8%) were pT0, 12 (15.0%) were downstaged to pT1, 17 (21.3%) were pT2 and 20 cases (25.0%) were pT3.

### Selection of cut-off scores based on ROC curve analysis

Cut-off scores were determined to be 50% for p53, 20% for VEGF, Bcl-2 and EGFR and 10% for APAF-1. Tumours with scores above the obtained cut-off values were considered positive for the expression of the protein. The corresponding AUCs (95% CI) are listed in Table 2. AUCs were the largest for EGFR (0.66 (0.54–0.78)) and VEGF (0.64 (0.51–0.77) indicating that the discriminatory power for complete response was the greatest for these two markers.

### Univariate analysis

**Figure 1:**
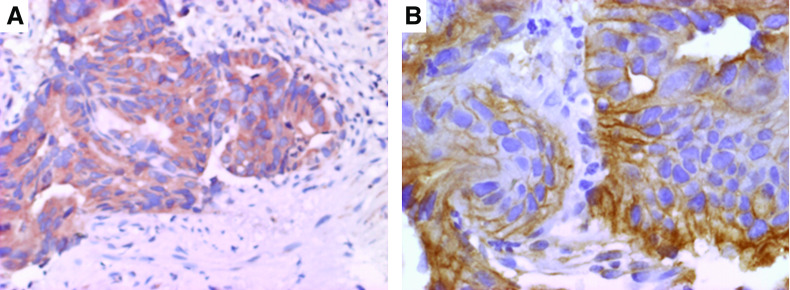
Representative immunostains of VEGF (**A**) and EGFR (**B**) from pretreatment rectal tumour biopsies.

The association of protein expression with complete pathologic response (Table 2) demonstrated that negative VEGF expression (*P*-value=0.004, OR (95% CI)=0.23 (0.09–0.63)) and EGFR positivity (*P*-value=0.003, OR (95% CI)=5.78 (1.85–18.07)) were significantly associated with complete tumour response after correction for multiple comparisons while p53, APAF-1 and Bcl-2 demonstrated no predictive ability for the outcome. Loss of VEGF expression was associated with more than a 4.3 times greater chance of complete tumour response compared with VEGF-positive tumours, while EGFR positivity resulted in a 5.78 times increased odds of complete tumour regression. Representative immunostains of VEGF and EGFR are illustrated in Figure 1.

### Multivariable analysis

**Figure 2:**
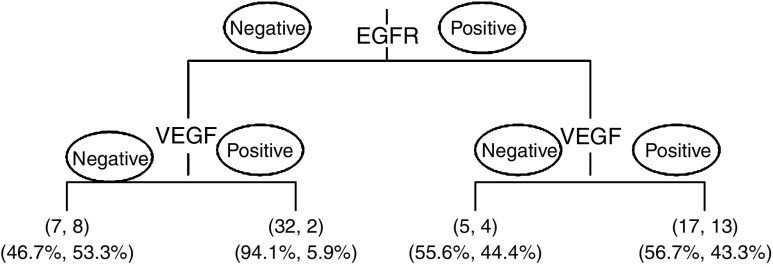
Decision tree summarising the frequency of complete tumour response with various multi-marker phenotype combinations. In first parentheses under each decision arm: number of patients with no complete response, number of patients with complete response. In second parentheses: proportion of patients with no complete response, proportion of patients with complete response.

VEGF and EGFR were entered into multivariable analysis. Eighty-eight tumours could be evaluated, of which 27 (31%) had a complete pathologic response. Loss of VEGF (*P*-value=0.009; OR (95% CI)=0.24 (0.08–0.69)) and positive EGFR (*P*-value=0.01; OR (95% CI)=3.82 (1.37–10.6)) both demonstrated independent predictive value for complete pathologic response. Multi-marker combinations of VEGF and EGFR were analysed and summarised in Table 3. Positive VEGF and negative EGFR expression were significantly associated with lack of complete pathologic response (*P*<0.001) compared with all other multi-marker combinations. The odds of complete response were 12.8 for VEGF-negative and EGFR-positive tumours compared with VEGF-positive and EGFR-negative tumours. Moreover, of the 34 EGFR-negative cases, which simultaneously had VEGF positivity, 32 (94.1%) had no complete pathologic response. These results are highlighted in Figure 2.

## DISCUSSION

The aim of this study was to determine whether pretreatment expression levels of five immunohistochemical markers including p53, Bcl-2, APAF-1, VEGF and EGFR could predict complete pathologic tumour regression in patients with advanced rectal cancer undergoing preoperative radiotherapy. Our findings indicate that VEGF and EGFR are independent predictive factors and their combined analysis is highly predictive of complete pathologic response.

Prognostic or predictive studies evaluating immunohistochemical markers, including EGFR, have often yielded irreproducible results. Several sources of discrepancy have been recognised as contributing to the conflicting reports in the literature between similar studies, including methodological differences such as various fixation protocols and antibodies (Atkins *et al*, 2004; McShane *et al*, 2005). The interpretation of immunoreactivity is underlined as a major source of contradictory findings (Goldstein and Armin, 2001; Resnick *et al*, 2004; Spano *et al*, 2005; Kim *et al*, 2006; Li *et al*, 2006). In order to avoid the use of predetermined and often arbitrarily set cut-off values, we have previously shown how ROC curve analysis in conjunction with a resampling procedure can be systematically used to evaluate the protein expression of immunohistochemical tumour markers (Zlobec *et al*, 2007a). Along with a reproducible semi-quantitative scoring system, ROC curve analysis is a powerful method for selecting cut-off scores to describe tumour marker positivity for a specific clinical endpoint, such as tumour response. In addition, we have recently demonstrated that assessment of staining intensity between independent observers results in low to moderate inter-observer agreement whereas the semi-quantitative evaluation of protein expression is highly reproducible between multiple observers and sufficient for providing the necessary information on the associations of protein markers with different clinicopathological endpoints (Zlobec *et al*, 2006a, 2007b, 2007c).

By applying ROC curve derived cut-off scores to the immunohistochemical markers in this study, we found that VEGF negative tumours were more than four times more likely to undergo a complete tumour regression than their VEGF positive counterparts. Complete pathologic response was nearly six times more likely in EGFR-positive tumours compared with EGFR-negative cases. Moreover, analysis of multi-marker phenotypes of VEGF and EGFR expression identified a subgroup of VEGF-positive and EGFR-negative tumours that were highly resistant to treatment.

VEGF is a potent angiogenic promoter required for the full execution of angiogenesis. VEGF receptor signalling has been shown to have effects on endothelial cell proliferation, migration, survival and vascular permeability (Roskoski, 2007). In addition, urokinase plasminogen activator, tissue plasminogen activator and matrix metalloproteinases are induced by upregulation of VEGF. These proteins function to degrade the basement membrane and extracellular matrix, providing a scaffold for proliferating, migrating and extravasating endothelial cells (Ferrara *et al*, 2003). The result of tumour angiogenesis is a newly formed vasculature with ‘leaky’, disorganised vessels, increased interstitial pressure and chaotic blood flow, which decreases the efficiency of radiotherapy (Willett *et al*, 2006; Roskoski, 2007). In 1971, Folkman (1971) hypothesised that blocking angiogenesis in tumours could be used a method of treatment for patients with cancer. Pharmacological agents designed to block VEGF or VEGF receptor signalling are currently in various phases of clinical trials and have demonstrating promising results (Kowanetz and Ferrara, 2006; Roskoski, 2007; Willett *et al*, 2007). The VEGF blockade results in ‘normalisation’ of the vasculature, decreased interstitial pressure and vascular permeability, thereby potentiating the effects of radiotherapy by increasing oxygen transport to tumour cells and facilitating the delivery of chemotherapeutic agents to the target tumour (Willett *et al*, 2006). In our study, VEGF positivity was strongly associated with a more radio-resistant phenotype.

In colorectal cancer, VEGF is associated with tumour aggressiveness, poor survival, local failure and the presence of metastatic disease (Giatromanolaki *et al*, 2006). Giralt *et al* (2007) demonstrated that VEGF positivity is an indicator of poor disease-free survival following preoperative radiochemotherapy, while Nozue *et al* (2001) described an association between post-treatment VEGF overexpression and distant metastasis. Qiu *et al* (2000) found no correlation between pretreatment VEGF expression and tumour response in 72 patients undergoing long-course neoadjuvant radiotherapy. In a previous work on the same, yet smaller series of patients, we not only identified VEGF but also Bcl-2 as significant predictors of tumour regression (Zlobec *et al*, 2005). Bcl-2-negative tumours experienced a partial or complete tumour regression more often than Bcl-2-positive cases. This protein, however, was not associated with complete tumour regression in this study, a discrepancy that is likely due to the different clinical endpoints evaluated in our previous and current works. These results suggest a possible role for Bcl-2 in tumour regression, which requires further elucidation. To our knowledge, the present study is the first to have evaluated complete pathologic response and pretreatment VEGF expression in rectal tumours. Our results clearly demonstrate that patients with VEGF-negative tumours before treatment should be considered candidates for preoperative radiotherapy.

Although the prognostic role of EGFR has been frequently investigated, only few studies have assessed the predictive value of pretreatment EGFR expression in preoperative radio- or radiochemotherapy. Recent reports are conflicting. While Giralt *et al* (2005) found a significant association between EGFR overexpression and a lack of complete pathologic tumour regression to preoperative radiotherapy, Bertolini *et al* (2007) reported no such result. The findings of our study indicate that pretreatment EGFR expression is an indicator of complete pathologic response and strongly supports the treatment of these patients with preoperative radiotherapy. Recently Jonker and co-workers assessed the value of the anti-EGFR therapy cetuximab on a large cohort of 572 patients with advanced EGFR-expressing colorectal cancers who failed to respond to previous chemotherapy, and found a significant improvement in overall survival in these patients. Anti-EGFR therapy may further improve clinical outcome in our series our EGFR-positive, and more radiosensitive patients treated with HDREB (Jonker *et al*, 2007).

Although our results, which indicate improved outcome in patients with positive EGFR, appear to conflict with the majority of reports in colorectal cancer, our findings are in line with previous studies in head and neck squamous cell carcinoma (HNSCC) using moderately accelerated or hyperfractionated accelerated radiotherapy. Eriksen *et al* (2005) investigated 803 patients randomised to 5 *vs* 6 fractions per week of radiotherapy. They found that high-EGFR tumours responded better to moderately accelerated radiotherapy compared with EGFR-low tumours, and determined that response to accelerated fractionation may be predicted by high EGFR expression in pretreatment tissue samples. Bentzen and co-workers performed IHC on 304 patients randomised to receive CHART *vs* conventionally fractionated radiotherapy. They concluded that there was a significant benefit from strongly accelerated CHART in patients with high EGFR expression and no benefit in patients with a low EGFR index (Bentzen *et al*, 2005). These results suggest that the predictive value of EGFR to radiotherapy may be dependent on the dose fractionation regimen.

In our combined analysis of VEGF and EGFR expression, both markers had independent value for predicting a complete pathologic tumour regression. Simultaneous VEGF-positive and EGFR-negative expression was associated with a lack of complete tumour regression in more than 94% of cases and a 12-fold-decreased odds of response compared with EGFR-positive and VEGF-negative tumours. A relationship between EGFR and VEGF has previously been established. Not only do both proteins share the same intracellular signalling pathways (Roberts and Der, 2007), but several preclinical studies have provided evidence for either direct or indirect angiogenic effects of EGFR signalling (Ciardiello *et al*, 2006). EGFR has additionally been reported to upregulate VEGF expression (Ciardiello *et al*, 2006). Recently Eriksen *et al* (2005) demonstrated that EGFR tyrosine kinase inhibitors decrease VEGF expression by both HIF-1*α*-independent and -dependent mechanisms. Although interrelated, the contribution of VEGF and EGFR to angiogenesis appears to arise through distinct mechanisms, thereby warranting the simultaneous blockade of both proteins for the treatment of patients with rectal cancer (Tabernero, 2007).

We acknowledge that preoperative HDREB remains an experimental approach that is presently being considered for a randomised trial. At the present time, different radiation schedules are used: in northern Europe, 25 Gy in five fractions (short course) is commonly applied, whereas 45 Gy in 25 fractions (long course) with chemotherapy is preferred in southern Europe and North America. Bujko *et al* (2006) randomised 310 patients with cT3 rectal cancer to 5 Gy × 5, followed by surgery or conventional preoperative 50.4 Gy plus bolus 5FU1leucovorin daily over 5 weeks, followed by surgery and reported similar local control and survival results. The ability to predict complete pathologic response or sensitivity to radiation based on IHC would have a significant impact on the selection of patients for preoperative radiotherapy or chemoradiation therapy schedules.

In this study, negative VEGF and positive EGFR expression were predictive of complete pathologic response to preoperative radiotherapy in patients with advanced rectal cancer. In addition, our findings have identified a subgroup of VEGF-positive and EGFR-negative tumours, which are more resistant to radiotherapy and should perhaps be considered candidates for innovative neoadjuvant combined modalities.

## Figures and Tables

**Table 1:** Clinicopathological characteristics of rectal cancer patients treated with high-dose-rate brachytherapy

	**Total**	**Frequency**	
**Clinicopathological feature**	***n***	***n***	**(%)**
Sex			
Female	104	74	67.3
Male		36	32.7
			
*Tumour grade (differentiation)*			
Well	92	21	22.8
Moderate		67	72.8
Poor		4	4.4
			
*Tumour response*			
Complete	104	33	31.7
Partial		35	33.6
None		36	34.6
			
*cT stage*			
cT1	102	0	0
cT2		2	2.0
cT3		96	94.1
cT4		4	3.9
			
*PT stage*			
pT0	80	31	38.8
pT1		12	15.0
pT2		17	21.2
pT3		20	25.0

**Table 2:** ROC curve-derived cut-off scores, area under the curve (AUC) and association of protein expression with complete pathologic response

**Cut-off score**	**AUC (95% CI)**	**Total (*n*)**	**No complete response, *n* (%)**	**Complete response, *n* (%)**	***P*-value**	**OR (95% CI)**
*p53*						
⩽50%	0.529 (0.39–0.66)	92	41 (70.7)	17 (29.3)	0.359	1.55 (0.61–3.96)
>50%			22 (64.7)	12 (35.3)		
						
*VEGF*						
⩽20%	0.64 (0.51–0.77)	89	12 (48.0)	13 (52.0)	0.004	0.23 (0.09–0.63)
>20%			49 (76.6)	15 (23.4)		
						
*Bcl-2*						
⩽20%	0.546 (0.41–0.68)	90	49 (72.1)	19 (27.9)	0.203	2.02 (0.68–5.99)
>20%			13 (59.1)	9 (40.9)		
						
*APAF-1*						
⩽10%	0.538 (0.41–0.67)	88	40 (71.4)	16 (28.6)	0.137	2.07 (0.8–5.38)
						
>10%			20 (62.5)	12 (37.5)		
						
*EGFR*						
⩽20%	0.66 (0.54–0.78)	90	40 (80.0)	10 (20.0)	0.003	5.78 (1.85–18.07)
>20%			22 (55.0)	18 (45.0)		

APAF-1=apoptosis protease-activating factor-1; CI=confidence interval; EGFR=epidermal growth factor receptor; OR=odds ratio; ROC=receiver-operating characteristic; VEGF=vascular endothelial growth factor.

**Table 3:** Multi-marker phenotype combinations of VEGF and EGFR in patients undergoing preoperative HDREB

**VEGF**	**EGFR**	**Total (*n*)**	**No complete response**	**Complete response**
Negative	Negative	15	7 (46.7)	8 (53.3)
Negative	Positive	9	5 (55.6)	4 (44.4)
Positive	Negative	34	32 (94.1)	2 (5.9)
Positive	Positive	30	17 (56.7)	13 (43.3)
	Total	88	61	27

EGFR=epidermal growth factor receptor; HDREB=high-dose-rate endorectal brachytherapy; VEGF=vascular endothelial growth factor.
